# The importance of theory and method: A brief reflection on an innovative program of research examining how situational factors influence physicians’ clinical reasoning

**DOI:** 10.1096/fba.2020-00109

**Published:** 2021-03-30

**Authors:** Alexis Battista, Abigail Konopasky, Steven J. Durning

**Affiliations:** ^1^ Henry M Jackson Foundation for the Advancement of Military Medicine Bethesda MD USA; ^2^ Uniformed Services University of the Health Sciences Bethesda MD USA

**Keywords:** clinical reasoning, context specificity, contextual factors, functional linguistics, scientific reasoning, self‐regulated learning, simulation, situated cognition

## Abstract

Clinical reasoning, a complex process that involves gathering and synthesizing information to make diagnostic and treatment decisions, is a topic researchers frequently study to mitigate errors. Scientific reasoning has several similarities with clinical reasoning, including the need to generate hypotheses; observe, gather, and interpret evidence; engage in the process of elimination; draw conclusions; and refine and test new hypotheses. However, researchers have only recently begun to take into consideration the role that situational factors (also known as contextual factors), such as language barriers or the lack of diagnostic test results, can play in diagnostic error. Additionally, questions remain about the best ways to teach these complex processes.

## 1 INTRODUCTION

In this article, we reflect on and highlight key findings from a program of research that sought to examine (a) the role of situational factors in physicians’ clinical reasoning and (b) how two increasingly popular learning strategies (i.e., live scenario‐based simulation and pre‐recorded video‐based simulations) influenced study participants’ clinical reasoning processes. We emphasize how our development and use of novel methods of analysis and the theoretical framework of situated cognition helped us to better understand the role of contextual factors, pointing to implications for the teaching and learning of scientific reasoning.

Mitigating diagnostic errors is an important component of efforts to improve healthcare quality and safety.^1^ *Clinical reasoning*, a complex process at the heart of a physician's practice that drives diagnostic and treatment decisions, includes the gathering and synthesizing of information, interpreting data (e.g., patient's responses to diagnostic questions, lab, or radiologic findings), and generating and refining hypotheses.^2^, ^3^ Situational factors (also known as contextual factors), such as language barriers, the patient suggesting an incorrect diagnosis or limited access to a patient's electronic health record, can be an unwanted source of variance leading to diagnostic error. This complexity can lead to *context specificity*.^4^ Context specificity refers to the phenomenon of seeing two patients with the same presenting symptoms and findings who have the same diagnosis and yet the physician comes to two different diagnostic decisions.^4^ As this new knowledge of clinical reasoning has come to the fore, calls to better understand how to measure and analyze context specificity and more specifically these situational factors have grown.

Scientific reasoning has several similarities to clinical reasoning. For example, *scientific reasoning* is often defined as a “problem‐solving process,” requiring individuals to generate hypotheses; observe, gather, and interpret evidence; engage in the process of elimination; draw conclusions; and refine and test new hypotheses.^5^, ^6^, ^7^ Furthermore, clinical reasoning and scientific reasoning are both the source of continuous efforts to determine how to best educate physicians or scientists given that they require extensive effort to develop and reach proficiency. For example, perspectives in clinical reasoning have viewed misdiagnosis as occurring due to limitations of physicians’ medical knowledge, cognitive biases, or how physicians organized and gathered information during a patient encounter.^8^ Similarly, earlier views on scientific reasoning emphasized the stages or states of scientific reasoning (e.g., initial state, goal state), scientists’ conceptions of scientific theory, and scientific problem‐solving processes.^6^ Thus, we believe that the challenges of understanding and teaching clinical reasoning have implications for the teaching and learning of scientific reasoning.

In this article, we reflect on our experiences employing *situated cognition theory* to study the influence and role of contextual factors (e.g., situational factors) in physicians’ clinical reasoning. Situated cognition theory posits that people come to know—or learn—through engagement in activities that are bound to specific social, cultural, and physical contexts.^9^ It differs from many traditional learning theories that frame learning as something that is transactionally “acquired” by learners or something that is “transferred from teacher to student.”

Situated cognition theory provided us with an opportunity not just to examine what participants’ knowledge was, but to examine how they interacted with a simulated patient within an outpatient context, and what choices they made in terms of questions they asked, physical exam maneuvers they opted to include or exclude, and additional studies to pursue. In these encounters, some of the important interactions to consider included those that can arise from physician factors (e.g., age, specialty, cognitive effort), patient factors (e.g., diagnostic suggestion, language proficiency), and encounter factors (e.g., functionality of electronic health record, time for patient visit, clinical context).^10^, ^11^, ^12^ We attended to the language participants used when interacting with the simulated patient, what information they gleaned from their interview and exam, and how they, in turn, formulated a diagnosis and treatment plan.

In keeping with situated cognition theory we also examined the role of learning context, specifically how two popular learning strategies used for teaching clinical reasoning (i.e., live scenario‐based simulation, pre‐recorded video‐based simulations) influenced study participants’ performance and clinical reasoning. Both contexts provide unique advantages for learning; however, they provide participants with different experiences. For example, live scenarios are socially rich encounters where participants engage “as if” they are in an actual clinical setting, where they have access to most of the information and data they would normally have when seeing actual patients. Live scenarios also present logistical challenges, such as the need for specialized faculty with simulation knowledge and added time. Conversely, video‐based simulations depict a clinical encounter and are easier to develop, employ, and distribute. However, while videos are set in a simulated outpatient setting and often portray a simulated patient and physician encounter, there are often limits to participants’ opportunities to actively engage with the depiction.

Lastly, situated cognition demands that we attend to the social, cultural, and physical aspects of learning, thus we did not rely solely on *outcome* measures of clinical reasoning, such as a final assessment or, in our case, a post encounter form whereby the participants documented their diagnostic and therapeutic decisions. We briefly discuss how our development and use of novel methods of analysis, such as functional linguistics^13^, ^14^ and self‐regulated learning microanalysis,^15^ helped us also better understand the *processes* of clinical reasoning and how contextual factors impact diagnosis and treatment. We believe that our findings are germane to scientific problem‐solving, especially systems biology approaches, for example, where complexity and situational factors are particularly evident.

## 2 BACKGROUND AND METHODS SUMMARY

This program of research, funded by the Joint Program Committee 1/Medical Simulation and Information Sciences Research Program (Award #NH83382416), had two primary aims: to examine the role of contextual factors on physicians’ clinical reasoning and the role that different types of simulation‐based learning contexts (i.e., live scenario‐based simulations and pre‐recorded video‐based simulations) play in supporting clinical reasoning. We used a mixed‐methods, experimental designs, and employed live scenario‐ or video‐based simulated sessions to elicit and analyze clinical reasoning. Participants—65 primary care or surgical physicians from three military treatment facilities—were recruited and randomly assigned to either the video or live scenario‐based condition.

To examine the impact of contextual factors, we used traditional *outcome* measures such as a post‐encounter form (PEF) which captured clinical reasoning outcomes (e.g., differential diagnoses, supporting evidence, primary diagnosis).^11^ We also employed functional linguistic analysis to examine cognitive *processes* in the presence of contextual factors. Functional linguistics, which uses grammatical structures like subject and object to offer insight into beliefs and emotions, helped us examine the inferences, cognitive processing, and affective reactions of participants.^16^, ^17^

Self‐regulated learning microanalysis was used to examine metacognition (which involves, among other things, being aware of, controlling, and managing one's cognition in pursuit of a task).^15^ Self‐regulated learning is a dynamic and cyclic process of thoughts, feelings, and actions that individuals engage in to achieve their goals.^5^ Microanalysis of self‐regulated learning processes involved focused questions examining physicians’ perceived challenges (e.g., what challenges they were aware of during the live or video simulation) and how they might modify or adjust their strategies to improve their learning and future performance (i.e., adaptive inferences^5^, ^15^).

Live scenario‐based simulations were conducted in a simulated setting designed to mimic an outpatient clinic. Study participants were provided with the same clinical tools (e.g., stethoscope, otoscope) as would normally be found in the clinical setting and were instructed to engage with the patient (portrayed by a trained actor) as if it was an actual clinical encounter. Pre‐recorded video‐based simulations depicted a clinical encounter between a patient and physician (both portrayed by trained actors) in a simulated outpatient clinic. Case type (i.e., typical presentations of new‐onset diabetes, unstable angina) and commonly reported contextual factors (e.g., language barriers, diagnostic suggestion) were controlled for all settings.

Following engagement in a live scenario‐ or viewing a video‐based simulation scenario, all participants sequentially:

Completed clinical reasoning measures (i.e., diagnosis and management plans; the PEF).

Engaged in a think‐aloud reflection (whereby an individual verbalizes their thought processes, without interruption, on a task—in this case, while viewing their own performance during a live‐scenario or rewatching the pre‐recorded video scenario),^18^ and.

Completed self‐regulatory microanalysis questions following their assigned simulation activity (see Battista et al., 2018 for detailed descriptions of scenarios and study procedures^19^).

## 3 SELECT RESULTS AND IMPLICATIONS

### 3.1 Implications of contextual factors’ effect on clinical reasoning

For contextual factors, a MANCOVA revealed significant differences in angina case clinical reasoning performance (as measured by the PEF) with and without contextual factors (*V(s)* = 0.72, *F* = 12.4, *df* = [6, 29], *p* <0.001), with univariate analyses indicating participants performed statistically significantly worse in cases with contextual factors. There were no significant differences in diabetes cases (see Konopasky et al. 2020b for more details on methods and findings^20^). These findings suggest that both context (e.g., specific situational factors; context specificity) and content (e.g., specific medical problems like angina; content specificity) are important for understanding clinical reasoning and reducing errors.

While both cases represent commonly encountered presentations for physicians, we speculate that the acuity of the unstable angina case may be what made it more difficult in the presence of contextual factors. In that case, the chest pain represents a life‐threatening condition and led to a number of potential management strategies, including sending the patient home with medication and instructions to limit activity and return if symptoms worsen until testing could be arranged, sending the patient directly to the emergency room, or directly admitting the patient. Analysis of participants’ reflections revealed that this aspect of the unstable angina case was nuanced. For example, participants weighed factors such as was the patient able to or likely to follow their instructions if care could or needed to be delayed? How quickly could additional testing be arranged? Perhaps either the uncertainty induced by the acute life‐threatening nature of the patient's presentation may have increased the mental effort enough that the introduced contextual factors overwhelmed participants’ cognitive capacity.

We believe this could also extend to scientific reasoning where, for example, it may be important to identify contextual factors (e.g., environmental, social) that can influence scientific reasoning processes. In other words, scientific reasoning performance may not only vary due to evidence evaluation, content area (e.g., biochemistry vs. genetics), procedural, or scientific knowledge, but also stem from situational factors (e.g., time for experiment, availability of laboratory equipment, comments made by others during an experiment; see Table 1).^21^, ^22^, ^23^ These results also indicate the importance of continuing research efforts to identify factors *other* than content or medical knowledge that contribute to establishing diagnoses as the findings were demonstrated in physicians with both limited and extensive practice experience.

**TABLE 1:** Comparison of factors that may influence clinical reasoning and scientific reasoning.

Situation factors influencing clinical reasoning	Situational factors that may influence scientific reasoning
Environmental factors include, workload, amount of time available to spend with the patient, access to/user interface of electronic health records, access to diagnostic testing capabilities.	Potential environmental factors, such as, **workload**, **time to conduct experiments**, **access to appropriate testing supplies**, access to **appropriate laboratory facilities or equipment**, **user interface** of scientific software.
Social factors, such as interruptions, language barrier between patient and physician, patient suggesting or insisting on an incorrect diagnosis, interactions with other healthcare professionals.	Social factors, such as **interruptions**, **communication** challenges, influence from or **suggestions made by colleagues**, **interactions with other laboratory team members**.

Note:

Bolded items indicate where situational factors that may influence clinical reasoning may be similar to scientific reasoning contexts.

### 3.2 The effects of contextual factors: Novel measures of clinical reasoning

In terms of novel linguistic measures, repeated measures MANOVA results revealed significant differences in some linguistic markers with and without contextual factors (*V(s)* = 0.22, *F* = 5.6, *df* = [3, 61], *p* < .01), with statistically significantly more cognitive process markers (phrases marking explicit reasoning like *seems* and *whether* or *not*) in think alouds of contextual factors cases.^17^ This demonstrates how linguistic tools can offer insight into the situated nature of the clinical reasoning process: here participants verbalized their cognitive processes more as they worked to make sense of the situation and the case with contextual factors.

Examination of cognitive processing terms related to learning or understanding (e.g., *think*, *explain*, *evaluate*, or *consider* ^13^) revealed that participants talked more about their learning or understanding when contextual factors were present, more often explicitly reflecting on their *thinking* or *considering*.^17^ Future work might explore how to co‐opt this verbalization of insight to support deeper metacognitive practices (i.e., how one thinks about one's own thinking) or as an assessment strategy to point to when an individual is *not* thinking about their thinking (see Konopasky et al., 2020a for more details on methods and findings^17^).

Additional linguistic subanalyses revealed more negative emotions (e.g., where participants thought aloud about their own or the simulated patient's stress or anxiety) when contextual factors were present.^17^ This suggests the need to be more mindful of the effects of contextual factors, including helping physicians identify and mitigate stress and anxiety during clinical encounters. Educational implications could include teaching physicians and scientists about the importance of situational awareness (e.g., how they organize their time, organize their workspace, prepare for a patient encounter) and how they may better manage their work environment.

In terms of self‐regulated learning microanalysis, we examined participant's adaptive inferences, which focus on how individuals modify or adjust their strategies to improve their learning and future performance. As part of a follow‐up descriptive analysis of aggregated group data for the microanalytic adaptive inference data, we found that 50% (*n* = 19) of the physicians did not believe they would need to do anything differently to improve their performance. A *post*‐*hoc* analysis revealed that approximately, 42% (*n* = 8) of the physicians who provided a “no change needed” response exhibited subpar performance.^16^ This suggests these participants inaccurately calibrated by over‐estimating that they would attain a higher performance than they actually did. The findings are important because they suggest that participants may lack adequate awareness of their own self‐knowledge or skill or have a poor understanding of the demands of the activity.^15^ Errors in self‐evaluation like this have implications for future tasks because these evaluations are used to prepare for future tasks. For instance, if an individual overestimates his/her performance and believes he/she do not have any limitations, he/she will not expend effort to alter his/her future attempts.

Both the linguistics cognitive processing of words measure and the SRL measure of adaptive inferences (i.e., what clinicians would do differently) show promise for helping instructors and learners determine not only when an intervention may be needed, but also what kind of intervention may be the most beneficial. For instance, learners could think aloud about a research problem while instructors listened for common cognitive processing markers followed by a written reflection about what the learners might do differently. Instructors could then offer an assessment of each learner's understanding of the problem. These three data points—the cognitive processing markers, SRL adaptive inference response, and instructor assessment—could then guide a conversation aimed at improving learners’ ability to assess when they may be out of their depth.

### 3.3 Differences in learning across simulated contexts

Regarding differences in simulated learning contexts and relying on self‐regulated microanalysis, physicians in the live scenario‐based simulation condition exhibited superior performance in clinical reasoning (as measured by the PEF), and a distinct profile of reflective judgments and cognitive processing (as measured by microanalytic and functional linguistic measures). Generally, the live scenario‐based simulation condition participants focused more attention on the aspects of the clinical reasoning process such as:

*Perceived challenges*: statistically significantly more physicians in the live scenario‐based simulation condition (*n* = 15, 78.9%) than video‐based simulation condition (*n* = 7, 36.8%) focused on the integration and synthesis of data as their primary challenge to accurately diagnose the case.^16^

*Cognitive processing*: linguistic analysis of the reflection activity revealed that individuals from the live scenario condition (*M* = 18.81, *SD* = 1.89) displayed a greater number of words reflective of higher levels of cognitive processing than those from the video condition (*M* = 16.84, *SD* = 3.49).^16^

Taken together, these findings offer a nuanced picture of how participants engaged in different levels and kinds of cognitive processing and reflection depending on simulation modality. One implication of these findings is that engaging in a live scenario‐based simulation enabled participants’ engagement in complex reasoning activities such as information synthesis, an activity central to clinical and scientific reasoning. Furthermore, rewatching one's own performance may lead to greater self‐awareness that prompts more insightful analysis of effective and ineffective actions. Participants in the live scenarios also had greater control over how the encounter unfolded, thus they were also likely able to relate more directly to their own actions. Thus, when possible, live scenario‐based simulations, where participants engage in problem‐solving, have the autonomy to determine their own actions and are provided with protected time to reflect, may be more robust learning environments for developing complex reasoning practices.

## 4 CONCLUSIONS

Our use of situated cognition theory as a theoretical framework led to several important insights. Our findings add to the growing body of literature seeking to explore and explain the underlying causes of misdiagnosis. For example, prior research has examined the role of error stemming from limitations in medical knowledge, cognitive biases, and exam strategies.^8^ These important perspectives focus primarily on factors that are internal to the individual physician. Our incorporation of situated cognition helped us focus on how factors outside the physician may influence clinical reasoning, such as language barriers and diagnostic suggestions. Taken together, our efforts help expand our understanding of the complex factors that make up and influence clinical reasoning.

We believe these approaches could also be used to better understand scientific reasoning and inform educational and policy decisions. For example, conceptions of scientific reasoning have also focused on individual‐level factors, such as scientific knowledge, theoretical assumptions, and knowledge of scientific processes.^6^ Incorporating a theory such as situated cognition could uncover the role that contextual factors play in scientific reasoning, potentially highlighting how certain kinds of factors—such as interruptions or lack of access to specific equipment—may influence scientific reasoning processes or outcomes.

Gaining a better understanding of these factors has potential applications to education and workplace policy. For example, if interruptions were to be identified as a major hindrance to accurate scientific reasoning efforts, organizations could enact policy (e.g., creating no‐interruption zones) and educational efforts (e.g., awareness training) in the workplace. Education stakeholders could choose to teach students about how to identify and combat the influence of contextual factors that are known to hinder scientific reasoning.

Given that situated cognition theory views knowledge as something that is emergent and gained through interaction, we also focused on how contextual factors affected the moment‐by‐moment cognitive processing of participants. Our efforts yielded several novel insights. Linguistic analysis shed light on how contextual factors led physicians to focus more on their own or the patient's negative emotions rather than their own thoughts or actions.^17^ These findings reveal how important it is to study the interactions between the individual and the context rather than singling out the individual or context. Additionally, linguistic analysis has the potential to be a powerful assessment tool because it offers a novel way of *listening for* language markers that may indicate when an individual is focusing on learning or gaining understanding (e.g., words like *think*, *explain*, *consider*). Those charged with teaching clinical or scientific reasoning could be trained to listen for these linguistic markers in authentic learning contexts, such as simulated or practice‐based settings.

Self‐regulated learning microanalysis helped us uncover a potential new source of error (i.e., lack of self‐awareness, lack of understanding of what is needed for clinical reasoning). Extensions of this analytic approach could involve employing self‐regulated microanalysis to monitor students’ progress over time whereby educators are able to evaluate and adjust their subsequent teaching efforts to address students’ deficits.

Lastly, our analysis of simulation‐based learning approaches suggests that not all simulations are the same. Research suggests that simulations are both practical (they can be used to protect learners or patients from harm, for example^24^), yet also ideal for facilitating a deeper understanding of complex concepts and the relationships between them^25^ and the processes of problem‐solving and decision‐making.^26^ Our findings focus on how different simulation modalities achieve or fail to achieve these goals—an important part of growing efforts to determine what works for whom and under what circumstances. Specifically, the live scenario‐based simulations enabled participants to engage in the important processes of gathering, integrating, and synthesizing data and in more complex cognitive processes. Notably, these are also essential to clinical reasoning. As our respective fields—clinical and scientific reasoning—evolve, and as our understanding of what clinical and scientific reasoning entail grows (in terms of the myriad of complex factors that influence them), stakeholders must also continue to examine which learning contexts are better suited to teaching and learning clinical and scientific reasoning.

## 5 DISCLAIMER

The views expressed in this paper are those of the authors and do not necessarily reflect the official position or policy of the US Government, Department of Defense, Department of the Navy, or the Uniformed Services University of the Health Sciences.

## CONFLICT OF INTEREST

Alexis Battista, Abigail Konopasky, and Steven J. Durning have no conflicts of interest to report.

## AUTHOR CONTRIBUTIONS

All authors collaborated together on the commentary. All authors offered substantive revisions and approval of this final version of the paper.
